# Adherence to medication for the treatment of psychosis: rates and risk factors in an Ethiopian population

**DOI:** 10.1186/1472-6904-12-10

**Published:** 2012-06-18

**Authors:** Menna Alene, Michael D Wiese, Mulugeta T Angamo, Beata V Bajorek, Elias A Yesuf, Nasir Tajure Wabe

**Affiliations:** 1 Regulatory Unit, Beker Pharmaceuticals General Business Plc, Lideta Kifle Ketema, Addis Ababa, Ethiopia; 2 Division of Health Sciences, School of Pharmacy and Medical Sciences, University of South Australia, Adelaide, Australia; 3 Clinical Pharmacy Unit, Pharmacy Department, College of Public Health and Medical Science, Jimma University, Jimma, Ethiopia; 4 School of Pharmacy, Graduate School of Health, The University of technology Sydney (UTS), Sydney, Australia

**Keywords:** Medication adherence, Antipsychotic, Compliant fill rate, Jimma

## Abstract

**Background:**

Medication-taking behavior, specifically non-adherence, is significantly associated with treatment outcome and is a major cause of relapse in the treatment of psychotic disorders. Non-adherence can be multifactorial; however, the rates and associated risk factors in an Ethiopian population have not yet been elucidated. The principal aim of this study was to evaluate adherence rates to antipsychotic medications, and secondarily to identify potential factors associated with non-adherence, among psychotic patients at tertiary care teaching hospital in Southwest Ethiopia.

**Methods:**

A cross-sectional study was conducted over a 2-month period in 2009 (January 15^th^ to March 20^th^) at the Jimma University Specialized Hospital. Adherence was computed using both a compliant fill rate method and self-reporting via a structured patient interview (focusing on how often regular medication doses were missed altogether, and whether they missed taking their doses on time). Data were analyzed using SPSS for windows version 16.0, and chi-square and Pearsons r tests were used to determine the statistical significance of the association of variables with adherence.

**Result:**

Three hundred thirty six patients were included in the study. A total of 75.6% were diagnosed with schizophrenia, while the others were diagnosed with other psychotic disorders. Most (88.1%) patients were taking only antipsychotics, while the remainder took more than one medication. Based upon the compliant fill rate, 57.5% of prescription fills were considered compliant, but only 19.6% of participants had compliant fills for all of their prescriptions. In contrast, on the basis of patients self-report, 52.1% of patients reported that they had *never* missed a medication dose, 32.0% *sometimes* missed their daily doses, 22.0% only missed taking their dose at the specific scheduled time, and 5.9% missed *both* taking their dose at the specific scheduled time and *sometimes* missed their daily doses. The most common reasons provided for missing medication doses were: forgetfulness (36.2%); being busy (21.0%); and a lack of sufficient information about the medication (10.0%). Pill burden, medication side-effects, social drug use, and duration of maintenance therapy each had a statistically significant association with medication adherence (P ≤ 0.05).

**Conclusion:**

The observed rate of antipsychotic medication adherence in this study was low, and depending upon the definition used to determine adherence, it is either consistent or low compared to previous reports, which highlights its pervasive and problematic nature. Adherence must therefore be considered when planning treatment strategies with antipsychotic medications, particularly in countries such as Ethiopia.

## Background

Medication adherence relates to a patient’s medication-taking behavior, and specifically refers to the extent to which a patient follows the mutually agreed treatment plan. Non-adherence to medication is known to be associated with poorer treatment outcomes, particularly in the management of chronic disease, yet numerous clinical studies have reported an average adherence rate of only 43.1% to 78.0% among patients receiving treatment for various conditions.

In the treatment and management of psychotic disorders, non-adherence to medication is particularly problematic. Antipsychotic medications are effective in treating psychiatric problems, including schizophrenia; however, the maximum benefit that a patient derives from these medications is highly dependent on their adherence to treatment [1]. The favorable rates of relapse prevention reported in controlled trials cannot usually be applied to everyday practice, due to poorer adherence in the ‘real world’ setting; as highlighted by Cramer and Rosenheck [2], outside of clinical trial settings, the average rate of adherence to antipsychotic regimens is only 58%.

Although non-adherence is a ubiquitous problem in medicine [3], the nature of schizophrenia and other psychotic disorders makes it especially difficult for patients to adhere to treatment [4]. First, schizophrenia is an illness in which insight into the condition, and therefore need for treatment, is more likely to be impaired compared to other illnesses [5]; this lack of insight has been shown to be associated with non-adherence to medications [6-8] and other psychosocial treatment [9]. Second, disorganization and cognitive impairment are additional symptoms of schizophrenia that interfere with medication management [10-12], particularly over the long-term given the chronic nature of the illness. In general, the more prolonged the medication treatment period, the lower the rates of adherence, and this has been reported in other chronic diseases [13]. Furthermore, the greater the exposure to treatment the more likely the patient is to experience side-effects, increasing the patient’s reluctance to adhere to treatment; unfortunately, the advent of atypical antipsychotics has not significantly reduced the potential for adverse drug events [14,15]. Finally, schizophrenia and its treatment (antipsychotics) are subject to stigma [16].

The impact of medication non-adherence on clinical outcomes in the treatment of schizophrenia is significant, as studies have shown that deviation from maintenance antipsychotic treatment leads to disease relapse, increased clinic and emergency room visits, and re-hospitalization [17,18]. Furthermore, mortality in persons with schizophrenia is two to four times greater than that in the general population [19,20], which is in part due to the out-right increased risk of suicide, but also to non-adherence to treatment for both the psychosis as well as other underlying physical illnesses [21]. Subsequently, this compromises the individual’s quality of life and function, and increases the economic burden to the health-system [22].

In Ethiopia, due to the under-resourced health-care system, medication non-adherence rates are potentially much higher, thereby contributing to a substantial worsening of disease, increased mortality, and increased health care costs [4]. Given the significance of this as a health issue and the scarcity of data to inform the scope of this problem in the local setting, the primary aim of this study was to evaluate adherence rates to anti-psychotic medications, and secondarily to identify possible reasons for non-adherence to medications, among patients with psychosis in Jimma University Specialized Hospital in Southwest Ethiopia.

## Methods

### Study design

A cross-sectional study was conducted over a 2-month period in 2009 (January 15^th^ to March 20^th^), on the internal medicine ward of the Jimma University Specialized Hospital (JUSH), which is the only referral hospital in the Oromia Regional State of South West Ethiopia. The adherence rate to anti-psychotic medications and identification of possible reasons for non- adherence was evaluated using patients self-reporting and pharmacy refill record. JUSH has four major in-patient wards: internal medicine (where psychiatric patients receive their treatment), surgery, pediatrics, and gynecology and obstetrics, and additionally provides ambulance/emergency services, pharmacy, outpatient services, blood bank, and diagnostics (e.g., laboratory, X-ray, ultrasound scanner, electrocardiogram).

### Patient selection

Since psychiatric patients receive their treatment in the internal medicine ward of the hospital, patients of the internal medicine ward were eligible for inclusion. All patients who were prescribed antipsychotics within a 3-month time period were identified and consecutive patients meeting selection criteria were approached about inclusion in the study. The sample was restricted to patients with a diagnosis of schizophrenia, schizoaffective disorder, mood disorder with psychotic features, or psychosis not otherwise specified. Patients must have received maintenance therapy for at least 3 months to be included in the study. Excluded were patients who were taking anti-psychotic medication for non-psychotic mood disorders (e.g. disorders or behavioral disorders secondary to other diseases)**,** patients who had started antipsychotic medication within the past 3 months, and patients below the age of 10.

### Data collection

Adherence to prescribed medication regimens was determined with a quantitative, structured questionnaire. Face and content validity of the questionnaires were assessed through in-depth discussion with three experienced faculty colleagues and five senior internists in the internal medicine department at the study site. In addition, the questionnaires were pre-tested to minimize ambiguity and ensure the completeness of data capture. The result of the pretest was not included in the final analysis. Patients who were refilling a prescription were interviewed with the developed questionnaire. Using this questionnaire, a patient who reported that they had never missed either a daily dose or the time of taking a dose was considered to be adherent.

Medication adherence was also assessed by the compliant fill rate (CFR) method [23]. CFR represents the proportion of total fills that are adherent, i.e., filled at time-appropriate intervals over a specified time period. Adherence was assessed by comparing the number of days of medication supply with the number of calendar days between fills. A prescription fill was considered adherent if it took place before the completion of the previous prescription and there was no more than 20% of the medication still with the patient [23,24]. For example, if a patient received 60 tablets on a prescription and was instructed to take one tablet a day, the fill would be considered adherent if the patient collected the subsequent prescription within 48–60 days of the previous one. An exception was when a prescription became invalid because of a change in therapy. In such cases, the medication was either prematurely or delayed in being filled, but the fill was considered adherent.

Medication profiles were also examined to calculate the number of scheduled oral daily medications and the total number of prescribed tablets or capsules prescribed per day. If a patient was prescribed more than one agent for a disorder, adherence rates for all medications were calculated and the agent with the highest non-adherence was recorded and used for data analysis.

### Sample size

Since there have been no previous study of medication adherence in Ethiopia, a prevalence rate (p) of 50%, confidence interval of 95% and margin of error (d) of 5% were used for sample size calculation. The sample size was calculated using the *[Z*^2^*p* (1-p)]/d^*2*^ formula, where Z is the standard normal confidence internal (1.96). Accordingly, the appropriate sample size was calculated as 384.

### Data analysis

Data were coded, checked for completeness and consistency, and analyzed using SPSS Version 16.0. Descriptive statistics were used to determine patient demographics, medication information, and adherence rates. The association between variables was calculated with Chi-square test of association and Pearsons r test where appropriate.

### Ethics

The proposal was reviewed and approved by the ethical clearance committee of Jimma University. The aims of the study were provided to potential participants and informed consent was obtained prior to inclusion in the study.

## Results

### Socio demographic characteristics of patients

Among three hundred and eighty four participants who were approached regarding the study, 336 were included, resulting in a response rate of 87.5%. Of the remainder, 48 either did not fulfill the inclusion criteria, or give written consent for inclusion in the study. The cohort was comprised of 54.8% males with a mean age of 35 +/− 11.9 years. The majority of the study subjects were Orthodox Christian by religion and 26.5% were unemployed (Table 1).

**Table 1:** Socio demographic characteristics of study participants

**S.No.**	**Socio-demographic**	**Characteristics**	**Frequency**	**Percent**
1	Gender	Male	185	54.8
		Female	151	45.5
2	Age (in years)	10-20	57	17.5
		21-30	109	33,6
		31-40	74	22.7
		41-50	58	18.0
		50+	37	8.1
3	Marital status	Never married	151	44.9
		Married	137	40.7
		Divorced	24	7.2
		Widowed	24	7.2
4	Living Condition	With family	300	89.9
		Living alone	19	5.5
		Others	17	4.5
5	Educational status	Diploma &above	62	18.3
		10-12 grade	117	34.7
		7-10 grade	71	21
		1-6 grade	33	9.6
		No formal schooling	53	13.2
6	Occupational status	Unemployed	89	26.5
		Private NGO	63	18.9
		Government	52	15.5
		Farmer	39	11.4
		Student	28	8.2
		Merchant	19	5.5
		Others	46	13.2
7	Religion	Orthodox	145	42.9
		Muslim	83	24.7
		Protestant	81	24. 2
		Catholic	13	3.7
		Others	14	4.6
8	Monthly income (In birr)	300+	108	32.1
		200-300	81	24.1
		100-200	72	21.5
		< 100	75	22.3

### Clinical characteristics of the patient

Amongst study participants, 75.6% were diagnosed with schizophrenia, 9.2% with schizoaffective disorder while the rest as mood disorder with psychotic features (11.9%) and psychosis not otherwise specified (3.3%). Most of the participants were taking only antipsychotics (88.1%), while 6.8% were taking antipsychotics and antidepressant, 3.9% antipsychotics and mood stabilizers and 1.2% a combination of antipsychotics, antidepressant and mood stabilizers. Participants attended for a medical checkup and prescription refill at 2 monthly intervals, during which time the pill counts, structured questionnaires and CFR analysis took place.

In relation to pill burden (in terms of the overall number of medications prescribed), the majority (74.0%) of patients were prescribed only one agent, 24.7% were taking two agents, and 1.4% were using three agents; the vast majority (96%) of doses were prescribed as once-daily dosing regimens.

In relation to the patients’ experiences of side-effects with their medications, 44.1% reported ongoing depression, 14.7% experienced weight gain, 8.8% had extra-pyramidal side-effects; nearly a quarter (23.5%) of patients reported experiencing multiple side-effects. Among those patients who experienced side-effects, when asked about what measures they had taken to avoid the side-effects, 87.2% responded that they did nothing, 3.2% of them stopped taking their doses, 4.6% informed their health professional/s, 4.1% told their family members, and 0.5% stopped going to work. Most (95.9%) of patients responded that they felt comfortable in openly discussing their medication issues with their health professional/s (e.g., pharmacists, doctors, nurses and others).

Most patients (72.6%) reported that they had no previous exposure to any *social*/ *recreational* drugs. Among those who had been exposed to these agents, khat (n = 83), cigarette (n = 28), and alcohol (n = 20), were most commonly reported by the patients. Among social/recreational drug users, 34% reported feeling depressed, 13.2% felt that their illness had worsened, and 18.9% reported feeling ‘different’ when they stopped using social/ recreational drugs.

### Adherence rate and factors associated with non-adherence

Adherence to antipsychotic medications was measured when patients presented for prescription (medication) refills and medical check-ups (outpatient clinic visits) every two months.

#### Patient’s self-reported adherence to medication

Approximately half (52.1%) of patients stated that they had *never* missed taking their prescribed medication, which included neither missing the daily dose outright nor missing the instructed time of dose administration. A small proportion (5.9%) of patients reported that they *sometimes* missed taking their medications in relation to either taking the daily dose outright and/or missing the instructed time of dose administration. Among those who reported that they had missed taking their medication, the most common reason (36.2%) for not taking doses was *forgetfulness* (Table 2).

**Table 2:** Patients reason for missing their anti-psychotic medication using self-report

**Reasons**	**Frequency**	**Percent**
Forgetfulness	59	36.2
Lack of information	17	10.5
Being busy	34	21.0
Decision to omit	20	12.4
Others	27	12.4
Both forgetfulness and being busy	7	3.8
Total	164	100.0

#### Compliant fill rate (CFR)

Based upon the compliant fill rate, 57.5% of prescription fills were considered compliant. Of the subjects with 100% CFR (n = 66, 19.6%), the number of females (n = 35, 23.2% of all females) was greater than the number of males (n = 31, 16.8%). Accordingly, at CFR of 25% (n = 98), CFR 50% (n = 99) and CFR 75% (n = 68), the number of male patients was slightly greater. When CFR was analyzed by marital status, 86 of 150 never married participants (57.3%) had a CFR <50%, whereas among the 137 married patients, 86 (62.9%) had a CFR < 50%. Distribution of rate of adherence by education status indicated that among the 66 patients with CFR = 100%, the majority (34.9%) had an educational status of 10–12 grades. The number of patients with educational status of diploma and above who had 100% CFR was 9.8%, compared to 9 of 45 (20%) who did not have any formal schooling. Conversely among the 61 patients who had an educational status of diploma and above, a large proportion (n = 46, 75.4%) had a CFR < 50% (Table 3).

**Table 3:** Number of adherence of patients defined by socio- demographic characteristics

**Socio-demographic Characteristic**	**Description**	**Compliant Fill Rate (CFR)**			
		**25%**	**50%**	**75%**	**100%**
Gender	Male	61	57	35	31
	Female	37	48	32	35
Age (in years)	10-20	20	12	12	12
	21-30	26	37	22	25
	31-40	20	25	12	17
	41-50	11	6	6	6
Marital status	Never married	38	48	29	35
	Married	38	48	28	23
	Divorced	11	3	3	3
	Widowed	6	3	6	5
Educational status	Diploma &above	23	23	9	6
	10-12 grade	28	37	29	23
	7-10 grade	23	18	14	15
	1-6 grade	6	11	3	12
	No formal schooling	17	11	8	9
Occupational status	Unemployed	20	291	12	28
	Private NGO	12	17	12	11
	Government	26	20	12	5
	Farmer	18	8	8	5
	Student	11	6	6	5
	Merchant	5	9	3	2
	Others	5	15	12	12
Religion	Orthodox	46	43	29	26
	Muslim	20	26	14	20
	Protestant	23	28	18	15
	Catholic	6	3	2	2
	Others	3	5	5	3
Monthly income (In Ethiopian Birr)	>300	18	22	15	15
	200-300	23	20	14	11
	100-200	26	22	14	9
	< 100	22	28	18	17
		9	12	6	14

Amongst social drug users, 80, 72.2 and 76.9% respectively had CFR < 50% (P = 0.05 for the comparison of all social drug users vs non-social drug users). When the number of medications taken was compared with the rate of adherence, it was found that, amongst patients with high adherence (n = 66, CFR = 100%), 53.5% were taking one medication and 44.2% were taking two medications. Furthermore, amongst patients with CFR = 75% (n = 68), 84.1% were taking just one medication. The number of medications and rate of adherence was significantly associated - as the number of medication increased, the patients’ adherence decreased (r = −0.12, p = 0.01).

One hundred eighty four patients (54.8%) took medications for more than a year and 152 for less than one year. When the rate of adherence was compared with the duration of antipsychotic drug use, it was found that those patients with a duration of maintenance therapy greater than a year had better adherence rate (r = 0.54, p = 0.04, Table 4).

**Table 4:** Association between rate of adherence and different variables

**Variables**	**P- value**	**Pearson correlation coefficient (r)**
Duration of medication	0.04	0.54
Side effect of medications	0.03	−0.19
Exposure to social drugs	0.05	−0.13
Number of different types of medication	0.01	−0.12

Based on the association of different variables and rate of adherence, there was no statistically significant association between age, sex, regime, income, educational, and occupational status of the patients (P > 0.05), but the duration of antipsychotic medication use, experiencing a side effect of medications, exposure to social drugs and the total number of medications currently taken did show a significant association with rate of adherence (Table 4).

## Discussion

### Methods of assessing medication adherence

Medication adherence describes the extent to which the patient continues the agreed treatment or intervention as prescribed. In other words, adherence can be defined as the degree of conformity between treatment behavior and treatment standard [4]. Understanding adherence to pharmacotherapies is crucial in clinical practice to ensure optimal clinical outcomes. There is no single measure accepted as the “gold standard” for assessing adherence, as each of the commonly employed methods have distinct advantages and disadvantages [25-27].

Frequently used methods of assessing participant adherence to pharmacological interventions include self-report measures: participant interviews, questionnaires, and diaries [27-31]. These methods are simple and inexpensive, but they are limited as they are subjective, crude and relatively inaccurate [25,32]. Although some suggest that patient-recall and refill history assessments are accurate enough (especially if they are performed in combination with other methods [33,34]), it is generally accepted that they substantially overestimate medication adherence [29,35]. Additionally, the ability of patient recall and refill history to detect changes in adherence is unknown [32].

Pill counts are inexpensive and are frequently utilized; whilst they provide information about the number of pills taken, it is difficult to determine actual medication consumption, as they rely upon the assumption that medications missing from the pill bottle were taken [36], and patients can intentionally or unintentionally manipulate this measure [25]. In addition, pill counts are laborious and rely upon accurate reporting dates for starting prescriptions. However, it has shown that pill counts can be more precise when carefully performed [25,32], and they can show both differences in patient adherence to therapy and reported the rate of adherence (from which we can detect non-adherence), in addition to measuring the changes in adherence across time [32]. Pill counts are regarded as being the most common way to assess adherence [37]. They are more accurate than self-report or refill history [38], but are tedious and difficult to administer [32]. Other measures such as electronic bottle caps are sophisticated and may be more accurate, but are costly [20], whilst biological methods (eg urine analysis and biomarkers) are precise measures of ingestion, but are not always available and practical [37].

### Medication adherence and factors associated with non-adherence

This study revealed that adherence to antipsychotic medications was low in our study population. Previously reported rates of adherence for anti-psychotic medications ranged from 25% to 75% [14,39-41], which is comparable to the reported adherence rate in this study, which showed that patients reported taking medications as prescribed and filled their medications at appropriate intervals just over half of the time. However, if we consider 100% CFR to represent the true rate of adherence, fewer than 20% of patients would have been considered adherent throughout the study period, which is quite low compared to these previous reports.

Given the consequences of antipsychotic discontinuation and haphazard antipsychotic use, the poor adherence rates demonstrated in this and other studies are troubling. Previous studies have reported that patients who discontinue antipsychotics may be two to five times more likely to relapse as other patients, leading to unnecessary suffering [22,42]. Robinson and colleagues [42] reported that 82% of first-episode patients experienced at least one relapse within 5 years of follow-up, and that patients who discontinued medication were five times more likely to relapse. It might be speculated that after experiencing one relapse, patients would be substantially less likely to discontinue medication, so our study is particularly noteworthy in suggesting factors that might contribute to non-adherence.

In our study over the half of patients reported that they had never missed either a daily dose or the time of taking a dose, and while over half of all prescriptions were filled in an adherent manner, only 19.6% of the study population was 100% adherent according to the CFR method. This demonstrates the fundamental controversy regarding which is the most appropriate method to measure medication adherence. Patients’ self-report may still represent an under-reporting of the magnitude of the problem [43], whereas methods such as CFR are less prone to manipulation, and may offer a better approximation of the true adherence status.

Whilst there was no association between adherence rates and age, gender, income, religion and educational status of the patients, we did find an association between the rate of adherence and duration of maintenance therapy. This is supported by some [14], but not other studies [11,43,44]. In a naturalistic sample, younger age is likely to be associated with a shorter duration of illness, which we observed to be associated with a lower rate of adherence. Two mechanisms may underlie this observation. The first involves patients early in the course of their illness being more willing to take risks to find out if they could remain well without medication, especially before they encounter repeated episodes of relapse. The second relates to a potential selection bias of our outpatient population, where patients with longer illness duration who attend clinic regularly are more likely to be compliant with their treatment. In our study, over half of our patients had received maintenance therapy for more than one year, and these patients had the highest rates of adherence. Investigators examining the course of schizophrenia have observed that positive symptoms show a modest improvement over time [45,46], and it may be that adherence improves along with the decline in these severe psychotic symptoms.

As with other studies [2,8,14], these results have shown an association between the quantity of medications taken and the adherence rate. This may be due to an association between an increasing number of medications with misunderstanding that may arise as a result of complex regimen and/or confusion of instructions from health professionals and health care givers.

Individuals noted to use social drugs (chat, cigarette, alcohol) were significantly less likely to be adherent to their antipsychotic medication. This finding is consistent with previous researches [45-50], and can be explained by the fact that social drugs themselves are a risk factor for several psychiatric manifestations. Similar research showed that active substance abuse has been found to have a nearly eight-fold higher risk of non-adherence [8]. These abusive substances may have an effect on the cognitive abilities of patients [51], which in turn may affect adherence.

Kozuki and Froelicher [51] demonstrated that comorbid situations may adversely affect medication adherence and prognosis. Since over three quarters of the participants in our study did not have additional medical illness, this was not assessed in this study, as the numbers were too small to draw accurate conclusions. Also, the effect of the type of antipsychotic medication (typical versus atypical) on the adherence rate was not assessed, as atypical agents were not available at the hospital during the study period.

In the present study, communication between the patients and the health professional was excellent, as over 95% responded that they felt comfortable in openly discussing their medication issues with their health professional/s. This clarity of communication is very supportive in overcoming a significant barrier to adherence, because when misunderstanding occurs, treatment becomes more complex and side effects are not managed.

Knowledge of side effects, dosage regimen and the name of their medications had an effect on adherence in this study, which replicates previous results [9,14]. Similar to other studies [1,16], we have shown that confusion and forgetfulness are the major obstacles in achieving adherence to a medication regimen.

### Limitation

The findings of this study should be interpreted with some acknowledgement of the limitations. Being a cross-sectional design conducted at a single university Hospital, the study findings might be non-representative of the broader patient population, particularly in view of the response rate. The self-report method used in this study to measure treatment adherence might substantially overestimate medication adherence, as it relies on patient recall. Furthermore, this approach might not fully identify all of the factors contributing to non-adherence, nor accurately measure incidence, or associations. Despite the above limitations, the study identifies issues for further in-depth investigation regarding medication adherence in psychosis in Ethiopia.

## Conclusion

Adherence to antipsychotic medications is relatively low. Pill burden, exposure to social/recreational drugs, and experiencing side-effects from medication may decrease adherence to treatment. Prescribers need to more closely focus on implementing treatment plans that patients understand and to which they agree and commit. The pharmacist should provide appropriate counseling on medications, emphasizing the importance of adherence, to both patients and their careers.

## Competing interests

The author(s) declare that they have no competing interests.

## Authors’ contributions

MA and NTW were principal investigators. NTW, MDW and BVB participated in the sequence alignment and write up of the manuscript. MTA and MA performed the data analysis. EA participated in patient identification and design of the study. MDW and BVB are also involved in language copyediting of the manuscript and revised the manuscript for intellectual content. All authors read and approved the final manuscript.

## Pre-publication history

The pre-publication history for this paper can be accessed here:

http://www.biomedcentral.com/1472-6904/12/10/prepub
